# Meeting of the Georgia Medical Society, of Savannah

**Published:** 1880-04

**Authors:** R. J. Nunn, Frank T. Lincoln

**Affiliations:** Prest. Ga. Med. Society; Sec. pro tem. G. M. S.


AT A MEETING OF THE GEORGIA MEDICAL SOCIETY, OF SAVANNAH, held March 23, 1880, the bills now before Congress to increase the powers of the National Board of Health were discussed, and as it is contrary to the by-laws of the society to pass resolutions upon matters of a public nature, it was decided that a paper be prepared expressive of the views of the individual members, to be signed by them and presented to the City Council, Cotton Exchange and the people of Savannah. In accordance with these instructions the following is offered:

We, the undersigned, members of the Georgia Medical Society, view with apprehension the extraordinary and dangerous powers proposed to be conferred upon the National Board of Health by the bills introduced in Congress by the Hons. Messrs. Harris and C. Young, and also that proposed by Mr. Acklen. The first section of Mr. Harris' bill provides: "That the National Board of Health, or in the intervals of its sessions, *its executive committee* (of five) *shall report* to the President of the United States whenever any place in the United States is considered *by it* to be dangerously infected with *contagious* or *infectious disease*, and that upon the official publication by the *President* of *such report*, the transportation of goods or persons from such a place into *another State* shall be unlawful, and all persons guilty thereof shall be liable to prosecution therefor in the circuit or district court of the United States for any district within which such goods or persons shall be transported, and any goods so transported shall be liable to be seized or destroyed, *unless such transportation shall be carried on in accordance with rules and regulations made by*

*the National Board of Health* and approved by the President, as in other cases. These rules *shall apply until the President shall proclaim such place no longer infected*, and in the meantime the board or its executive committee shall report to him weekly, in writing, the sanitary condition of the place in question."

Mr. Acklen's bill provides for the establishment of *quarantine stations* upon all avenues of approach to *any place declared to be infected* UPON INFORMATION furnished by the National Board of Health, at which points all freight or persons and their vehicles of transportation if *starting from or destined for a point beyond* the *borders of the State*, shall be inspected by the agents of the National Board of Health. After they shall have been declared by *such agents* free from infection, any *person* or *authority whatsoever* who shall *interfere* with the free passage of such person or freight or vehicle of transportation, on the charge that they are infected or dangerous to the public health, shall, on conviction before a *United States court*, be fined $1000 and imprisoned one year.

We consider the provisions of these bills as dangerous, revolutionary and useless should they become laws. We protest most strenuously against conferring such extraordinary powers upon any National Board of Health, believing that such powers could be used to destroy our commerce and liberties, and are subversive of the rights of States.

We earnestly appeal to the municipal authorities, the merchants and the public of Savannah, to unite in a protest against the enactment of such laws.

We would further suggest the holding of a convention in which all the Atlantic and Gulf States should be requested to participate, in order to frame a *bill* wherein our *commonweal* should be *explicitly* provided for.

Signed:

R. J. Nunn, M. D., Prest. Ga. Med. Soc.

Henry Le Hardy, M. D., Rec. Secretary, Treasurer and Librarian of G. M. S.

Jno. M. Johnston, M. D., Cor. Secretary G. M. S.

Jas. B. Read, M. D.

I. P. S. Houston, M. D.

T. B. Chisholm, M. D.

Robt. P. Myers, M. D.

T. J. Charlton, M. D.

Benj. S. Purse, M. D.

Wm. Duncan, M. D.

Benj. F. Sheftall, M. D.

Jno. D. Martin, M. D.

Jas. S. Morel, M. D.

Frank T. Lincoln, M. D.

J. C. Le Hardy, M. D.

J. T. McFarland, M. D.

W. G. Bullock, M. D.

H. Burford, M. D.

B. W. Hardee, M. D.

B. S. Herndon, M. D.

There are three members dissenting, one not replying.

Office President Ga. Med. Society,
SAVANNAH, *March* 27, 1880.

The above is a complete roll of the members of the Georgia Medical Society at present in the city of Savannah.

R. J. NUNN,

Prest. Ga. Med. Society.

FRANK T. LINCOLN,
Sec. *pro tem.* G. M. S.

A true copy. Attest: FRANK T. LINCOLN, Sec. *pro tem.* G. M. S.

